# Imaging of the Effect of Alcohol-Containing Media on the Performance of Hypromellose Hydrophilic Matrix Tablets: Comparison of Direct Compression and Regular Grades of Polymer

**DOI:** 10.3390/pharmaceutics12090889

**Published:** 2020-09-18

**Authors:** Nihad Mawla, Sarah Hanley, Karl Walton, Waseem Kaialy, Tariq Hussain, Adam Ward, Jonathan Brown, Barbara R. Conway, Peter Timmins, Kofi Asare-Addo

**Affiliations:** 1 Department of Pharmacy, University of Huddersfield, Queensgate, Huddersfield HD1 3DH, UK; nihad.mawla@hud.ac.uk (N.M.); A.Ward@hud.ac.uk (A.W.); b.r.conway@hud.ac.uk (B.R.C.); 2 Drug Product Science and Technology, Bristol Myers Squibb, Moreton, Merseyside CH46 1QW, UK; sarah.hanley@bms.com (S.H.); jonathan.brown@bms.com (J.B.); 3 EPSRC Future Metrology Hub, University of Huddersfield, Huddersfield HD1 3DH, UK; K.Walton@hud.ac.uk; 4 School of Pharmacy, Faculty of Science and Engineering, University of Wolverhampton, Wolverhampton WV1 1LY, UK; w.kaialy@wlv.ac.uk; 5 The Wolfson Centre for Bulk Solids Handling Technology, Medway School of Engineering, University of Greenwich, Kent ME4 4TB, UK; t.hussain@gre.ac.uk

**Keywords:** hypromellose, alcohol, dissolution, tomography, magnetic resonance imaging, tribo-electrification

## Abstract

As the ingestion of drug products with alcohol could have adverse effects on the release of drugs from dosage forms, it is important to understand the mechanisms underpinning the influence on drug release by evaluating the effect of alcohol-containing media on the behaviour of pharmaceutical excipients. In this work, the effect of hydroalcoholic media containing up to 40% *v*/*v* absolute ethanol was evaluated, employing both the regular (CR) and direct compression grades (DC) of hypromellose. X-ray microtomography (XµT) and magnetic resonance imaging (MRI) were used as complementary techniques in determining the influence of the media composition on the ability of the CR and DC polymers to form and evolve the gel layer that controls drug release. Particle and powder properties of the polymer were characterised to determine any relationship to performance in hydroalcoholic media. Triboelectrification results showed the CR grade formulation to charge electropositively whereas the DC grade charged electronegatively. The flow properties also showed the DC grade to have a superior flow as compared to its CR counterpart. Differences in particle morphology between the grades influenced charging and flow behaviour of the powders; however, it did not seem to impact significantly either on the mechanical strength or the drug release properties of the compacted formulation using the model drug propranolol HCl. XµT and MRI imaging were successfully used as complementary techniques in determining the gel layer/hydration layer thickness measurements as the layer developed, as well as following ingress of hydroalcoholic media and its impact on the dry core. The result showed that although differences were present in the gel layer thickness potentially due to differences in particle morphology, this also did not impact significantly on the dissolution process, especially in acidic and hydroalcoholic media.

## 1. Introduction

Palladone^®^, a hydromorphone hydrochloride extended release narcotic analgesic, was removed from the US market by the Food and Drug Administration (FDA) after clinical studies indicated an interaction when ingested with alcohol. The plasma concentration of hydromorphone exceeded therapeutic levels and potentially reached hazardous levels because of the alcohol-induced failure of the extended release component [1,2]. This loss of controlled release property phenomenon, termed dose dumping, is defined as the unintended fast release of drug with consequent undesired rapid absorption from extended release formulations [3]. This alert issued by the FDA identified the potential influence of alcohol on extended release matrices, and studies on the impact of hydro-alcoholic solutions on extended release matrix systems followed [4].

The oral drug delivery route still remains the route of choice over other delivery systems [5]. Modifying the kinetics of drug release from orally administered solid dosage forms can be attained by formulating the drug to include a hydrophilic polymeric matrix, using swellable polymers such as hypromellose (hydroxypropyl methylcellulose, HPMC), poly(ethylene oxide) and hydroxypropylcellulose. When the hydrophilic polymer is exposed to water or biological fluids, it becomes hydrated, swells and forms a gel layer (the zone between the erosion front and the swelling front) around an initially dry core, which can delay the diffusion of an incorporated drug from the polymeric matrix [6,7,8,9]. This gel layer hydration process is dynamic, with the gel layer growing over time due to further inward migration of fluid, as well as swelling of the gel layer and erosion of the gel layer due to shear forces in the environment in which the dosage form sits (agitation in an in vitro test, peristaltic forces in vitro). The rate of drug release from these matrices could be influenced, in addition to formulation variables, by the composition of the surrounding medium it is exposed to after ingestion, such as pH, enzymes, electrolytes and surfactants [2,10]. These parameters are therefore important to evaluate as dose dumping could have detrimental effects.

Coating systems for matrix tablets using guar gum and ethylcellulose have been explored as a way of overcoming dose dumping [3,11,12]. Jedinger et al. analysed the impact of alcohol on film coatings and hot-melt extruded pellets [13,14]. The authors proposed pore blocking as a novel approach for developing a safe and effective extended release dosage form that reduced risks of dose dumping in alcohol [15]. Nep et al. also explored starch-free grewia polysaccharide in comparison to the native grewia gum and found extraction of starch to improve the blending process and increase the resistance of the polysaccharides to alcohol damage [16]. The same authors also explored the effects of hydroalcoholic media on an extracted sesamum gum polysaccharide [17]. Ethanol has been reported to not have any dose dumping repercussions on the kinetics of drug release from hypromellose incorporated matrices [2,18]. Missaghi et al. investigated the impact of hydro-alcoholic solutions on different grades of controlled release hypromellose and also found no dose dumping to occur in contact with alcoholic medium, although the rheological and textural features of the hydrated polymer were affected [4]. Smith et al. studied release from several controlled release formulations in different percentages of hydro-alcoholic media. The authors observed that 5% content of alcohol in the medium did not significantly alter the kinetics of drug release but there were differences at higher alcohol ratios, with drug release being enhanced at those levels for capsules but inhibited from most tablets [19].

The readily commercially available hypromellose (CR grade) has a narrow particle size range and is usually used for wet granulation [20] and for dry granulation, e.g., roller compaction [21,22]. The introduction of a directly compressible (DC) grade of hypromellose [23,24] has overcome issues of poor flow associated with the CR grade. Van Snick et al. reported that tablets, compacted to a standard porosity, produced similar strength tablets [25]. Ervasti et al. [20] reported a study using the continuous manufacturing process to conclude that the CR grade of hypromellose controls the release rate of the model drug ibuprofen better than its DC counterpart [20]. 

This work therefore aimed to evaluate the effect of hydroalcoholic media on the gel/hydration layer of the CR and DC grades of the hypromellose using X-ray microtomography (XµT) and magnetic resonance imaging (MRI) and its impact on the release of a model drug propranolol hydrochloride. We also sought to understand the critical characteristics of the CR and DC polymers with regard to their comparative mechanical properties (including flow and charging propensities) to identify any relationships to their interactions with alcohol. To the best of our knowledge, this is the first systematic report combining such techniques to evaluate the CR and DC grades of hypromellose. 

## 2. Materials and Methods

### 2.1. Materials

Hypromellose 2208, 100,000 MPa.sec grade Methocel^®^ CR and DC2 (which will now herewith be referred to as CR and DC) and pregelatinised starch (Starch 1500^®^) were gifts from Colorcon (Dartford, UK). Particle size analysis showed CR to have a d_10_ value of 30.9 µm, d_50_ of 87.5 µm and d_90_ of 217.0 and DC to have a d_10_ value of 50.0 µm, d_50_ of 125.0 µm and d_90_ of 268.0 µm, respectively, using a laser diffraction particle size analyser (Sympatec, Clausthal-Zellerfeld, Germany) according to the methodology detailed in Asare-Addo et al. [26]. Propranolol HCl (PPN) purchased from TCI Chemicals, (Zwijndrecht, Belgium) was used as the model drug. Microcrystalline cellulose (PH102) (MCC) and magnesium stearate were purchased from Merck (Darmstadt, Germany) and Peter Greven, (Manchester, UK), respectively. Fumed silica (Aerosil^®^ 200) was purchased from Degussa, (Roussillon, France). Potassium chloride (Acros Organics, Loughborough, UK) and hydrochloric acid (Fisher Scientific, Loughborough, UK) were used in the preparation of pH 1.2 media and potassium phosphate monobasic-white crystals (Fisher BioReagents, Loughborough, UK) and sodium hydroxide (Fisher Scientific, Loughborough, UK) used in the preparation of pH 6.8 media. The hydro-alcoholic solutions (5 and 40% *v*/*v*) with either pH 1.2 or pH 6.8 phosphate buffer were made using absolute ethanol (Fisher Scientific, UK). All materials were pharmacopoeial grade or analytical grade laboratory reagents.

### 2.2. Micrometric Properties of CR and DC Powders

Scanning electron microscopy (SEM) (Jeol JSM-6060CV, Jeol, Welwyn Garden City, UK) operating at 20 kV was used to determine the morphology of the CR and DC polymers and help to explain how this could potentially impact drug release performance. The true densities of the formulation blends (Section 2.3) and the pure CR and DC polymers were determined using the Micromeritics Accupyc II pycnometer 100 (Micromeritics, Norcross, GA, USA). The bulk and tap densities of the CR and DC formulation blends and CR and DC polymer alone were also determined. A volume of 10 g of each formulation blend was weighed and gradually introduced into a 100 mL measuring cylinder. The bulk volume was noted and then the cylinder tapped till the volume was constant and recorded. This allowed the calculation of the bulk and tapped density, which is the ratio of the weight of powder to the bulk or tapped volume, respectively [27].

Powder flow was studied using an Erweka Granulate Flow tester (GTL type, Heusenstamm, Germany) and was determined from the flow time. Around 10 g of each sample was poured through a 6.0 mm nozzle coupled to the equipment. The time taken to discharge each powder was recorded. During flow testing, particles adhered to the inner surfaces of the stainless steel hopper (200 mL). Particle *%* adhesion was calculated from mass difference by subtracting the final amount recovered (post testing) from the initial amount of sample loaded into the stainless steel hopper.

It is important to evaluate the development of a static charge by pharmaceutical materials during handling as this can have beneficial or detrimental effects on the mixing process, which can result in ordered mixtures or risk of segregation [26,28,29,30]. It was important in this work to create homogeneous mixtures for tableting to ensure that comparisons between different materials were reliable. The charging properties of the CR and DC powders were analysed using a recently developed novel approach at the Wolfson Centre at the University of Greenwich. This novel method allows the detection and measurement of charge distribution on the charge sign basis in a population of particles. The experimental apparatus consists of a single non-contact electrostatic inductive sensor (probe), a charge amplifier unit, a national instrument (NI) data acquisition equipment and personal computer for data recording and processing. A sample of each powder was fed into the cylindrical sensor with the help of vibratory feeder and conveyed toward the sensor by gravity in a vertical direction [31,32,33]. Special care was taken by considering the adhesion property of particles with the wall of the sensor. The inner tube was replaced in order to remove any deposits, impurities or surface charge that may have been present on the surface from a previous test. Each sample was analysed using a fresh sample. Humidity and temperature were controlled in the laboratory (50% RH, 22 °C). 

### 2.3. Tablet Manufacture and Mechanical Strength Testing

Flat-faced, round CR and DC tablet matrices with a diameter of 8 mm and a target weight of 250 mg, containing 20% *w*/*w* of the model drug propranolol HCl, were formulated according to Table 1. All the excipients were sieved prior to blending to ensure that agglomerates were broken up to better provide for uniformity of blend. A Turbula^®^ (Type T2C, Muttenz, Switzerland) blender was used to blend the drug and excipients indicated in Table 1 (except the colloidal silica and magnesium stearate) at 49 rpm for 8 min. The colloidal silica and magnesium stearate were then added to the blend and mixed for a further 2 min. The tablet compacts were made using the Piccola 10 station automated tabletting machine with SMI software (Riva, Ludlow, UK) at a compression force of 15 kN and stored in a tightly closed screw cap glass/plastic container at room temperature, for at least 24 h, prior to testing. 

Breaking force was determined using the PharmaTest (Hainburg, Germany) hardness tester (*n* = 10 compacts for both CR and DC). The thickness and diameter of CR and DC compacts were measured using a digital calliper. This allowed for the determination of tablet porosity between the CR and DC compacts to be determined using Equation (1). Pure polymer compacts of the CR and DC grades were also prepared.

(1) Tablet porosity=[1−[tablet weighttablet volumetrue density of powder]]× 100

### 2.4. X-Ray Microtomography (XµT) Analysis of Compacted Formulations

The XµT was conducted in two ways. The first set of experiments, which involved using the instrumentation to determine the homogeneity of the CR and DC compacts, was conducted according to the methodology reported by Laity et al. [34]. In brief, double-sided adhesive tape was used to mount the formulated compact onto a sample stage, after which a set of 1583 projections was collected using the XμT, (Nikon XT H 225, Nikon Corp., Tokyo, Japan), using a tungsten target, with 90 kV accelerating voltage and 80 μA gun current. The projection images were reconstructed using CT-Pro and examined using VG Studio 2.1 software [34]. 

The second set of experiments involved hydrating, at room temperature (~22 °C), CR and DC compacts in a sealed glass vial containing 10 mL of either one of the four media (i.e., pH 1.2 (0% absolute ethanol), pH 1.2 (40% absolute ethanol), pH 6.8 (0% absolute ethanol) or pH 6.8 (40% absolute ethanol)) for up to 7 h. After the 7 h period, the media were decanted gradually to ensure the hydrated compact was not disturbed. The vial was sealed again to avoid evaporation and then mounted onto the sample stage of the XμT instrument (Nikon XT H 225, Nikon Corp., Tokyo, Japan). A set of projections were obtained and images reconstructed using CT-Pro and examined using VG Studio 2.1 software. The cross-sectional images generated from the CR and DC formulation were based on the differential absorbance of X-rays between materials that have differing electron densities [34,35,36]. After the reconstruction, the calliper option was used to mark and measure the gel layer thickness of the compacts. These were present as a result of the different densities and the measurements were taken from the centre of the hydrated compacts.

### 2.5. Magnetic Resonance Imaging (MRI)

**Figure 1:**
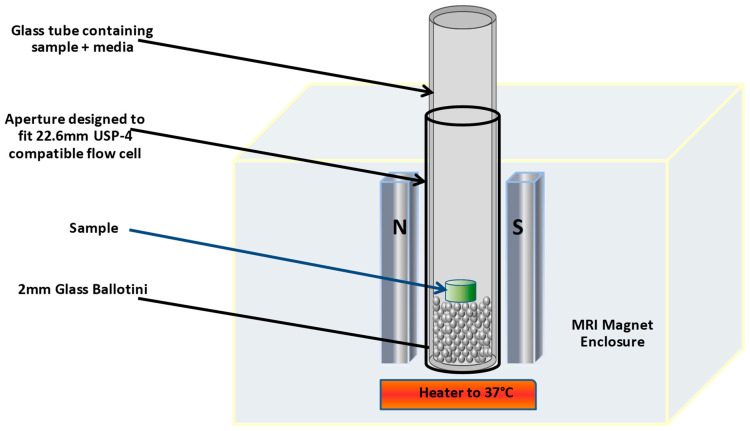
MARAN-i low-field magnetic resonance imager, with static cell set-up.

An Oxford Instruments MARAN-i low-field bench-top 20 MHz magnetic resonance imager (Figure 1) was used for the imaging studies. A static cell set-up was used; a glass test tube (26 mm wide) containing clear glass ballotini (diameter 2 mm, Marienfeld, Germany) to a depth of approximately 1 cm was further filled to a depth of approximately 5 cm with either 0.1 M HCl, 0.1 M HCl + 40% absolute ethanol, pH 6.8 phosphate buffer (0.05 M potassium phosphate) or pH 6.8 phosphate buffer (0.05 M potassium phosphate) + 40% absolute ethanol, which was inserted into the vessel holder in the magnet and maintained at 37 °C for the duration of the imaging. A dosage form was placed into the static cell and aligned horizontally on the surface of the glass ballotini. A standard 2D Spin Echo Multi-Slice Imaging mode was used, with a slice thickness of 3 mm and an in-plane resolution of either 128 × 128 or 256 × 256 µm. The echo time (Te) was set at 6 ms, and repetition time (Tr) was varied from 500 up to 1500 ms. The number of scans was also varied, from 2 to 4, which, together with the various resolutions and repetition times, changed the scan times from 256 to 768 s (scan time = 2D resolution × Tr × number of scans).

Images were obtained every 15 min in order to correlate with time-points for dissolution and tests were performed in duplicate; however, additional images every 3 and 5 min were obtained for the first 90 min portion of the run in order to provide more detailed information. The images obtained were analysed using IDL Virtual Machine Image Analysis software and the dry core area and swollen layer areas (total area—dry core area) were determined as area in mm^2^. 

### 2.6. Hydro-Alcoholic Dissolution Studies

The dissolution medium used in the determination of PPN release was 900 mL of pH 1.2 (0% absolute ethanol), pH 1.2 (5% absolute ethanol), pH 1.2 (40% absolute ethanol), pH 6.8 (0% absolute ethanol), pH 6.8 (5% absolute ethanol) or pH 6.8 (40% absolute ethanol) equilibrated to 37 ± 0.5 °C. An automated USP dissolution apparatus II (paddle method) with a paddle stirring speed of 100 rpm was used, with samples being withdrawn at time intervals of up to 720 min using a peristaltic pump. The PPN concentrations in the withdrawn samples were determined by UV spectrophotometry at 289 nm. 

### 2.7. Dissolution Parameters and Similarity Factor (f2)

The mean time for PPN to dissolve under in vitro dissolution conditions, the mean dissolution time (MDT), depicted as Equation (2) [37,38,39,40], was used to describe dissolution as it is a model-independent method that is suitable for dosage forms having different mechanisms of drug release. The mean dissolution rate (MDR) and dissolution efficiency (DE) were also calculated. The dissolution efficiency is the area under the dissolution curve up to a certain time *t*, expressed as a percentage of the area of a rectangle described by 100% dissolution in the same time *t* (Equation (3)) [40].

(2) $$MDT=\frac{\sum_{j=1}^{n}t_{j}\;\Delta M_{j}}{\sum_{j=1}^{n}\Delta M_{j}},$$

where *j* is the sample number, *n* is the number of dissolution sample times, $t_{j}$ is the time at midpoint between $t_{j}$ and $t_{j-1}$ and $\;\Delta M_{j}$ is the additional amount of drug dissolved between $t_{j}$ and $t_{j-1}$.

(3) $$DE=\frac{\int_{0}^{t}y\times dt}{y_{100\;}\times t}\times 100,$$

where *y* is the drug percent dissolved at time *t.*

Similarity between the PPN release profiles for both the CR and DC compacts was determined using similarity factor *f_2_* (Equation (4)) [41,42].

(4) $$f2=50\;log\left\{{\left[1+\frac{1}{n}\sum_{t=1}^{n}w_{t}(R_{t}-{T_{t})}^{2}\right]}^{-0.5}\times \;100\right\},$$

where *n* is the number of pull points for tested samples; $w_{t}$ is the optional weight factor; $R_{t}$ is the reference assay at time point *t*; $T_{t}$ is the test assay at time point *t*.

The similarity factor was calculated using PPN release profile from the CR compacts in pH 1.2 (0% absolute ethanol) as the reference for PPN release profiles from the CR compacts in pH 1.2 (5% absolute ethanol) and pH 1.2 (40% absolute ethanol). For CR compacts in pH 6.8 (0% absolute ethanol) was the reference and PPN release profiles from the CR compacts in pH 6.8 (5% absolute ethanol) and pH 6.8 (40% absolute ethanol) were compared to it. To evaluate differences between the CR and DC samples, PPN release from the CR compacts in both the pH 1.2 and pH 6.8 hydro-alcoholic media were used as the reference and their corresponding DC counterparts compared to it. *f*2 values ranging from 50 to 100 indicate similarity between the two profiles. The closer the *f*2 value is to 100, the more similar or identical the release profiles. Values of *f*2 less than 50 indicate dissimilarity between two dissolution profiles [43,44]. The similarity factor was determined for both the CR and DC compacts in all the media studied (pH 1.2 (0% absolute ethanol), pH 1.2 (40% absolute ethanol), pH 6.8 (0% absolute ethanol) and pH 6.8 (40% absolute ethanol)) up to the 85% PPN release and then using the whole drug release profiles also to determine if this impacted on the similarity profiles.

### 2.8. Kinetics of Drug Release

The Korsmeyer–Peppas equation [45,46] was used in the determination of the kinetics of drug release. Although the application of this equation can give limited insights into the exact release mechanism, it can be more informative than applying the Higuichi equation, hence its use in this current studies [46]. For cylinders, *n* values of up to 0.45 suggest Fickian diffusion whilst values of *n* above 0.89 suggest case II transport occurring (and perhaps with hydrophilic matrix systems suggesting a significant contribution from erosion of the hydrated layer as a factor in the drug release mechanism). However, an *n* value between these two suggests anomalous transport occurring, as reported in numerous studies [46,47], perhaps indicating multiple parallel mechanisms of drug release in operation. In these sets of experiments, we have determined the kinetics of the drug release for both the CR and DC compacts in all the media studied (pH 1.2 (0% absolute ethanol), pH 1.2 (40% absolute ethanol), pH 6.8 (0% absolute ethanol) and pH 6.8 (40% absolute ethanol)) up to the 60 % PPN release and then using the whole drug release profiles to determine if this impacts on the kinetics.

## 3. Results and Discussion

### 3.1. Physical Properties of Formulation and Tabletting

**Figure 2:**
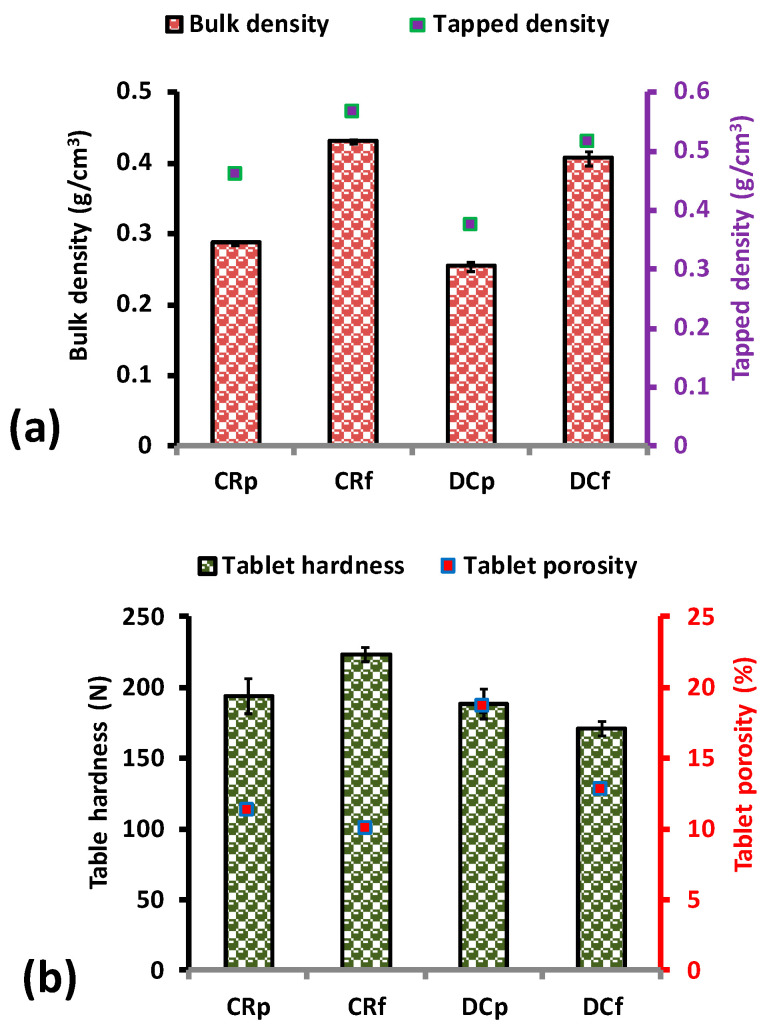
Bulk and tapped densities of polymer and formulated blends of CR and DC (**a**), tablet compact properties of hardness and porosity and for the polymers and formulated blends of CR and DC (**b**). Note: CRp is the pure CR polymer, CRf is CR formulation as detailed in Table 1, DCp is the pure DC polymer, DCf is the DC formulation as in Table 1. “Pure” in this context means the actual polymer with no active pharmaceutical ingredient (API) or additives.

The CR and DC polymers had the same true density (1.317 ± 0.001 g/cm^3^). The inclusion of the other excipients to form the formulation blends resulted in blend true density values of 1.393 and 1.397 g/cm^3^ for the CR and DC formulation blends, respectively. This increase was attributed to the contribution of the other excipients blended with the polymer as in Table 1. Figure 2, which depicts the properties of the CR and DC polymers as well as their formulation blends, shows an increase in the bulk and tapped density of the formulated blends over their counterpart pure CR and DC polymers. The CR samples in both cases had relatively higher bulk and tap densities than that of their DC counterparts. It was also interesting to note that there was a reduction in the porosity of the CR and DC formulated compacts as compared to their pure compacts (Figure 2). This may be attributed to better packing enabled by the various excipients used. CR compacts were harder as compared to DC equivalents, most likely due to a relatively higher solid fraction. This can also account for the differences in the compactability of the two grades as well as the differences in their particle size [48]. 

**Figure 3:**
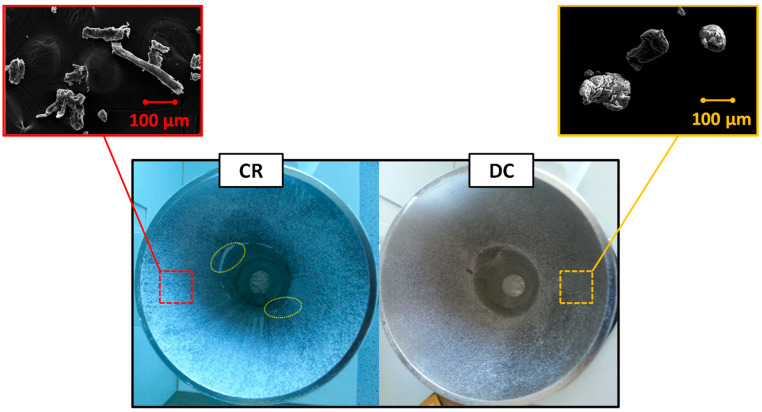
Stainless steel container post flow rate analysis. Square inserts are SEM images of the recovered pure polymer powders. Circular lines on the CR funnel image show the accumulation of powder at the funnel bend, which was not observed for the DC samples. Note: These are just for the pure polymers.

**Figure 4:**
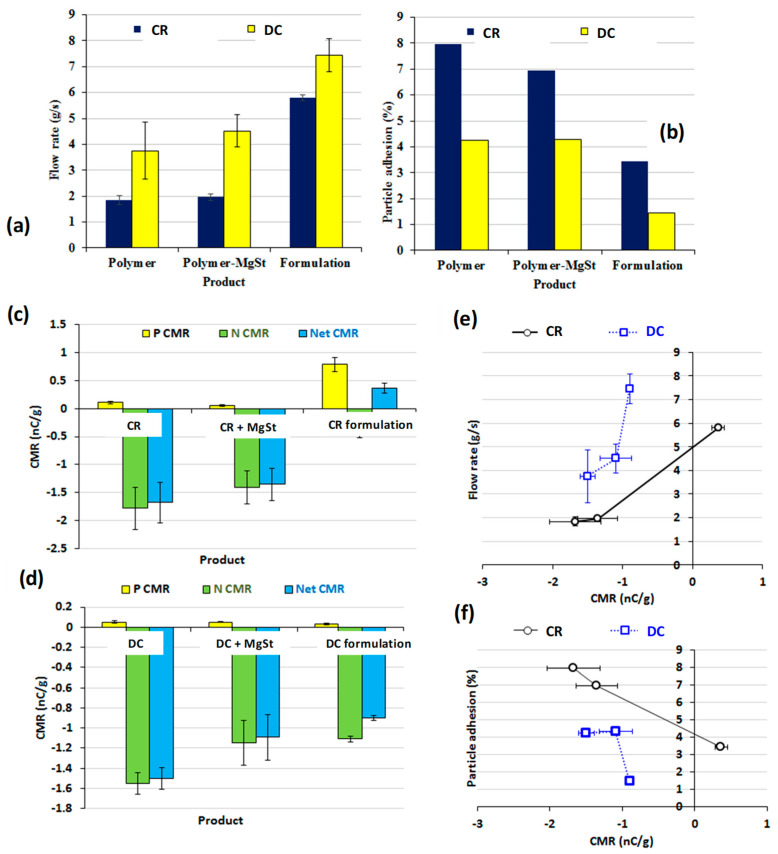
Powder flow rate (g/s, mean ± SD, *n* = 5) (**a**), % particle adhesion to stainless steel walls post flow rate analysis for powders under investigation (**b**), positive charge to mass ratio (P CMR), negative charge to mass ratio (N CMR) and net charge to mass ratio (Net CMR) of the CR material (**c**), positive charge to mass ratio (P CMR), negative charge to mass ratio (N CMR) and net charge to mass ratio (Net CMR) of the CR material (**d**), plot of the flow rate of CR and DC samples under investigation against the net-CMR (**e**) plot of the % particle adhesion of CR and DC samples under investigation against the net-CMR (**f**).

SEM images (Figure 3) showing the stainless steel container post flow analysis confirmed that CR and DC comprise different particle shapes, with the CR being irregularly shaped and the DC being rounder and agglomerated [20]. This morphology suggests that DC could have considerably better flow characteristics than CR and this was demonstrated to be the case (Figure 4a). The addition of magnesium stearate to the CR and DC blend appeared to slightly improve the flow properties of both polymers, although this improvement was not statistically significant (*p* > 0.05). The CR and DC formulation blends, however, had considerably better flow properties than the hypromellose itself, regardless of its grade (Figure 4a). The particle adhesion was relatively low (<10%, *w*/*w*) for all powders investigated (Figure 4b). DC, however, resulted in considerably lower adhesion (4.2% versus 8.0%) to the stainless steel walls (Figure 3). The addition of excipients reduced the adhesion of both polymer particles, which would be expected on the inclusion of magnesium stearate (Table 1). There was also a significant difference in the Carr’s index values for the formulated CR and DC blends (24 and 21%, respectively) over their pure CR and DC polymers (38 and 32%, respectively).

Both grades of hypromellose (CR and DC) have predominately electronegative charge behaviours, with no significant difference in their overall net-CMR (Figure 4c,d). The blends containing magnesium stearate were also electronegatively charged, although the addition of magnesium stearate slightly decreased the overall net-CMR. Blends containing CR demonstrated bipolar charge behaviour with an overall electropositive net-CMR, whereas those containing DC produced a predominately electronegative charge. Regardless of charge sign, powders containing CR had a lower absolute net-CMR than those containing HPMC DC (Figure 4c,d). The addition of excipients as well as processing parameters have been reported to have an effect on the charging properties of APIs [26,28,29,32,33,49]. Plotting the flow rate of different samples under investigation against the net-CMR showed powder flowability to decrease with the increase in net-CMR (Figure 4e). Powders with a relatively higher net-CMR demonstrated higher particle adhesion (Figure 4f).

### 3.2. X-Ray Microtomography

**Figure 5:**
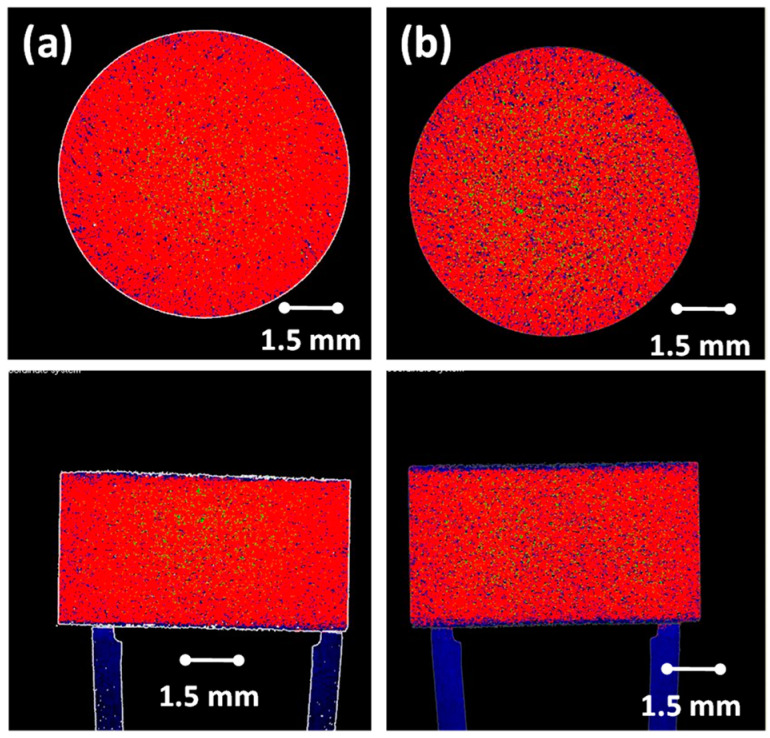
X-ray micro-tomographic images of sagittal and transverse sections through (**a**) CR and (**b**) DC formulation compacts. Images depict the homogeneity of the formulation mix.

The reconstructed X-ray micro-tomographic images of the CR and DC formulated compacts are depicted in Figure 5. The cross-sectional images generated from the CR and DC formulation showed that, despite the morphological and particle size differences between the CR and DC polymers, the distribution of the constituents within the formulations (as reported in Table 1) in both cases was homogeneous (Figure 5). XµT has been used to report on density variations in tablets and porosity and morphology in granules [50,51,52]. The more porous structure of DC compacts is also evident [53].

**Figure 6:**
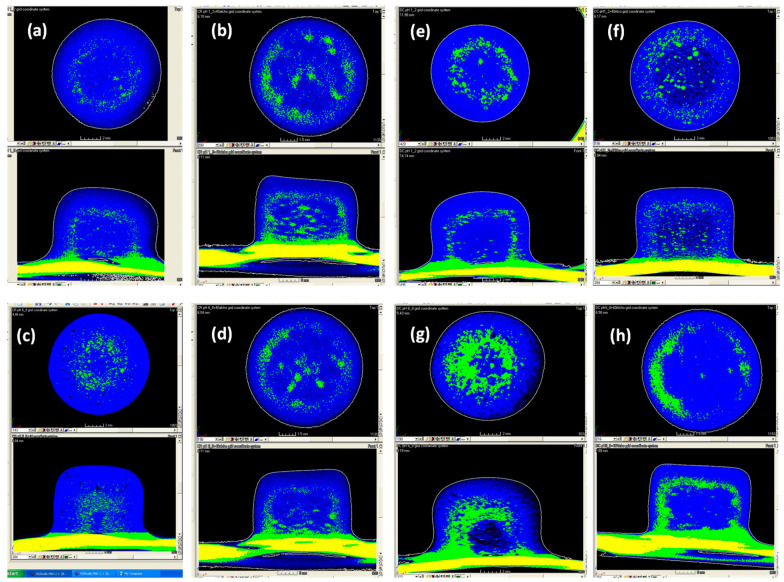
X-ray micro-tomographic images of the sagittal and transverse sections of the HPMC CR formulations in (**a**) pH 1.2 (0% absolute ethanol), (**b**) pH 1.2 (40% absolute ethanol), (**c**) pH 6.8 (0% absolute ethanol) and (**d**) pH 6.8 (40% absolute ethanol) for up to 7 h. X-ray micro-tomographic images of the sagittal and transverse sections of the HPMC K100M DC formulations in (**e**) pH 1.2 (0% absolute ethanol), (**f**) pH 1.2 (40% absolute ethanol), (**g**) pH 6.8 (0% absolute ethanol) and (**h**) pH 6.8 (40% absolute ethanol) for up to 7 h.

Figure 6 represents the tomographical images of the CR and DC formulated compacts hydrated for 7 h in pH 1.2, pH 6.8 and their corresponding alcoholic media. The images revealed that the gel layer thickness for the CR and DC formulations was similar in the acidic media (1.63 ± 0.9 and 1.68 ± 0.04 mm, respectively) (Figure 6a,e). It was, however, interesting to note that the thickness of the gel layer was significantly reduced in the presence of alcohol (pH 1.2 with 40% *v*/*v* absolute ethanol content) for both the CR and DC compacts (1.18 ± 0.12 and 0.97 ± 0.14 mm, respectively) (Figure 6b,f). This was the same in pH 6.8 media (CR = 1.78 ± 0.10 and DC = 1.61 ± 0.09 mm, respectively) and the pH 6.8 media with 40% *v*/*v* absolute ethanol content (CR =1.42 ± 0.15 and DC = 0.94 ± 0.06 mm, respectively) (Figure 6c,d,g,h). It is important to note that gel layer thickness was measured in the axial direction. It has been reported, and was observed here also, that swelling occurs to a greater extent in the axial direction than the radial direction and it can provide for a more reproducible estimate of gel layer thickness evolution [18,54,55,56,57,58,59]. Several authors have reported that alcohol can also inhibit the development of the gel layer of polymeric matrices [10,18,60]. These differences in the gel layer thickness with alcohol content may potentially impact the mechanism and kinetics of drug release and may occur due to an interaction between the media and hypromellose tablet, resulting in different media penetration rates [18].

### 3.3. Magnetic Resonance Imaging

**Figure 7:**
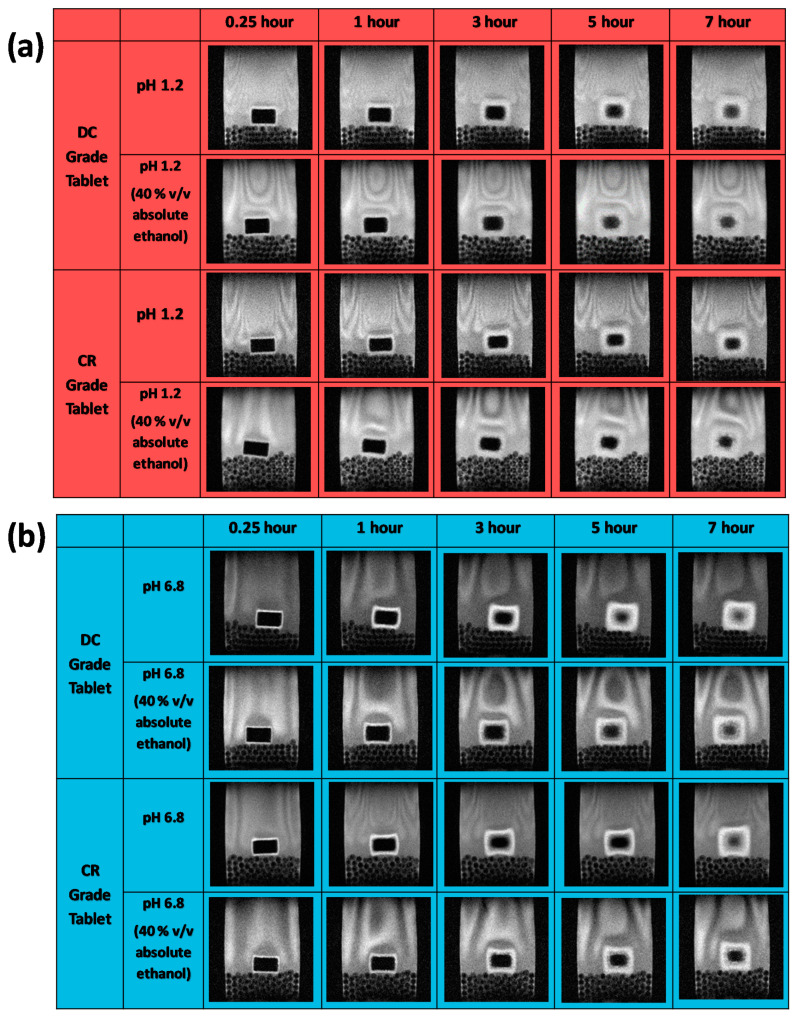
Magnetic resonance imaging of CR and DC formulations in (**a**) hydrochloric media with and without alcohol, (**b**) phosphate buffer media with and without alcohol.

**Figure 8:**
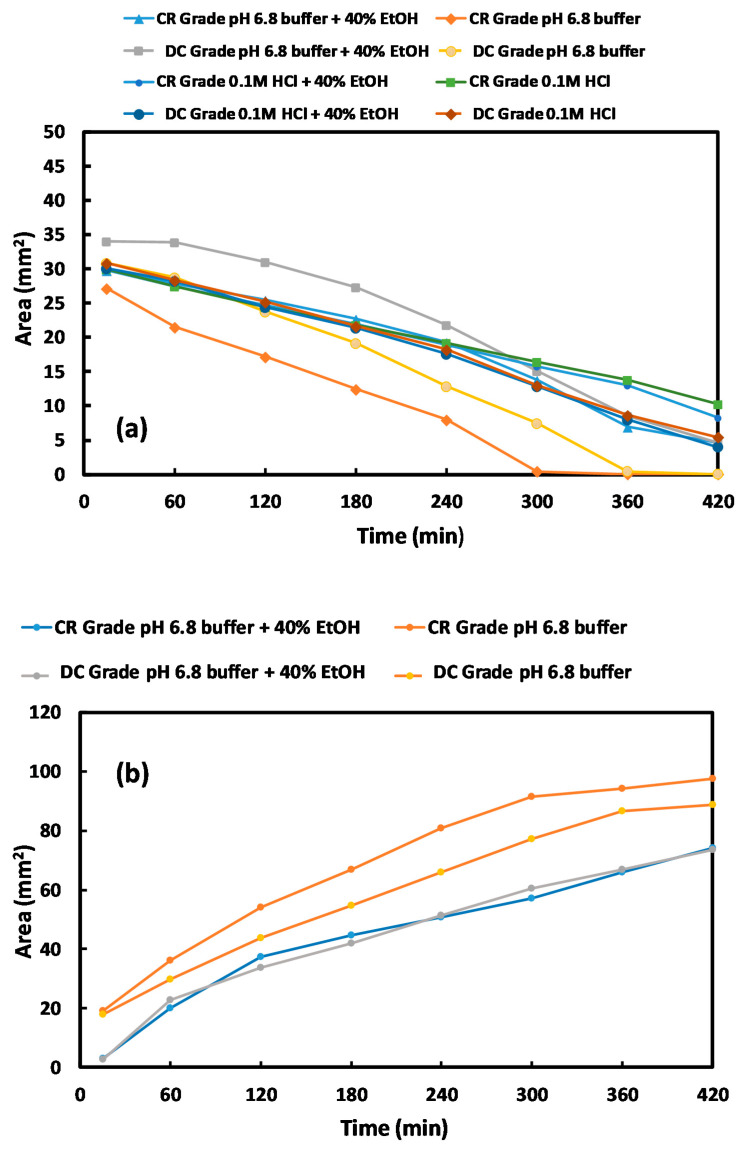
(**a**) Diminution of the mean dry core area of CR and DC formulations using MRI images, (**b**) development of mean gel area of CR and DC formulations using MRI images (experiments conducted in pH 6.8 and pH 6.8 (40% absolute ethanol)).

Figure 7 depicts magnetic resonance images of the CR and DC compacts in pH 1.2 and pH 6.8 media with and without alcohol at 40% *v*/*v*. The dry cores of the compacts for both grades (which appear black in the images as they do not contain media) in pH 1.2 and pH 1.2 (40% absolute ethanol) similarly reduce in size up to 4 h, after which time the reduction was slightly faster for the DC grade. There were, however, no differences between behaviour in the media with and without alcohol (Figure 8a). At low pH, a lack of a clear demarcation of the erosion front (the layer in contact with the dissolution media) meant that the gel areas could not be measured using the contour tool provided by the software for analysis due to poor contrast in the MRI images with these media. At pH 6.8, however, the reduction in size of the dry core for both formulations was significantly faster (F1 analysis) in pH 6.8 buffer alone, in the absence of alcohol. At pH 6.8, the dry core dimensions decreased more rapidly for the tablets containing the CR grade compared to those containing the DC grade, irrespective of the presence or absence of alcohol (Figure 8a). 

**Figure 9:**
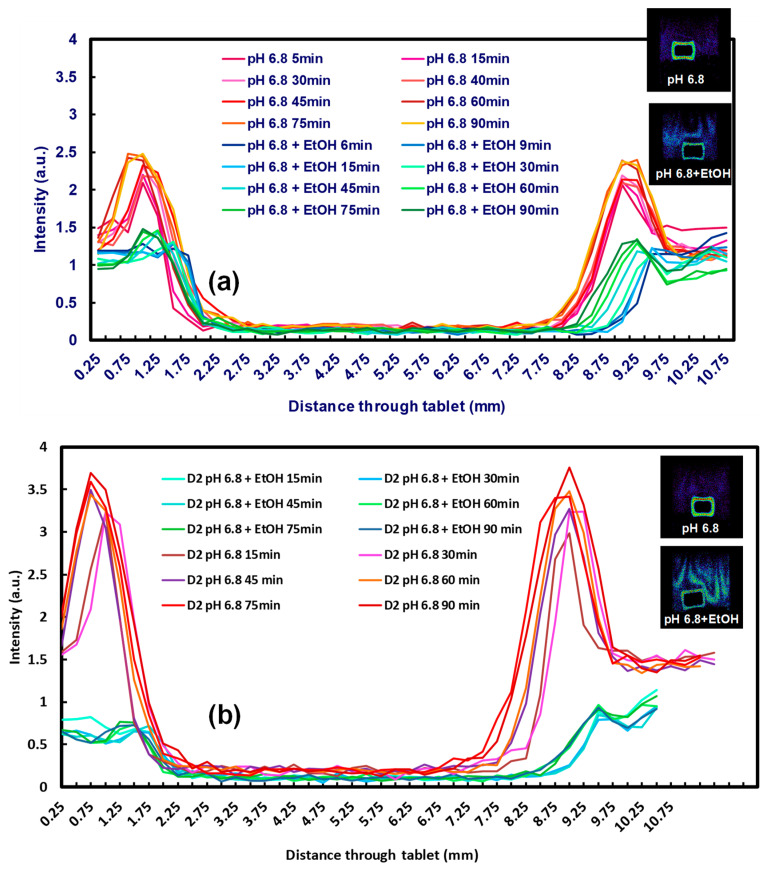
One-dimensional profiling of (**a**) CR grade compact in pH 6.8 medium every 5 min for 90 min in the presence and absence of alcohol (128 matrix, 500 ms, 6 ms), (**b**) DC grade tablet compact in pH 6.8 every 5 min for 90 min in the presence and absence of alcohol content (128 matrix, 500 ms, 6 ms).

At pH 6.8, gel layer swelling was faster for both formulations (CR and DC) in the absence of alcohol (Figure 8b). There was, however, no significant difference between the measured gel areas when hydration occurred in the presence of alcohol. Overall, there was a faster reduction in the dry core and growth of the gel area for the CR grade. In addition, the impact of alcohol was only measurable at pH 6.8. Further analysis of the gel layers (depicted as the insert in Figure 9) indicated a difference in the appearance of the gels, with that formed in the alcoholic media being less coherent and more unevenly patchy.

The 1D profiling of the compacts (Figure 9) also depicts a difference in intensities between DC and CR grades, confirming that the inclusion of alcohol affects gel formation, which may impact drug release. MRI has been used to produce quantitative and qualitative data previously. Using ultra-fast MRI, if was found that the concentration gradient of the media within the gel layer is relatively small and that more swelling occurred axially than radially [57]. Laity et al. [61] also found MRI to be a useful tool in determining the movement of the hydration front in polymeric matrices [61]. MRI has also been previously used to study different swelling dynamics in hydrophilic matrices [62,63]. The results obtained from the MRI studies aligned with the tomography data, showing that it was a complementary technique for determining the effects of hydroalcoholic media on gel growth. 

### 3.4. The Effect of Alcohol on Drug Release

**Figure 10:**
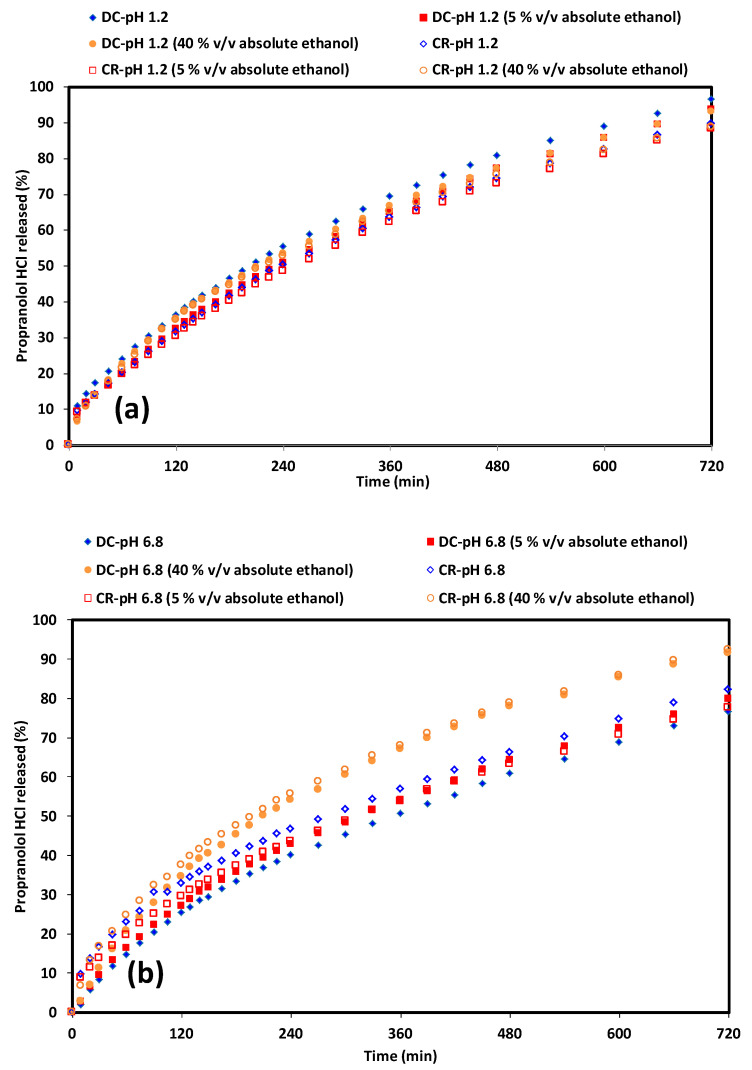
PPN release from CR and DC compacts (**a**) at pH 1.2 with differing alcoholic content, (**b**) pH at 6.8 with differing alcoholic content.

The inclusion of alcohol (at 5 and 40% *v*/*v* absolute ethanol content) had little impact on the release of PPN from the CR and DC compacts in acidic media (Figure 10). There was no marked initial release from either polymer. This was also found by Nep et al., 2017 where a similar result was reported for theophylline release from hypromellose and grewia polysaccharides [16]. Drug release at pH 1.2 was similar in the absence or presence of alcohol for both CR and DC grades (Table 2). Interestingly, when the CR and DC release profiles were compared, the similarity value at pH 1.2 was 64.4. Particle morphology and hydration kinetics may have contributed to this observation.

As discussed earlier in relation to the MRI results, the reduction in the dry core was faster for CR than DC compacts. Similar values were found for the CR and DC compacts in pH 1.2 medium in the presence of a small amount of alcohol (5% *v*/*v*), whereas this increased to 86.2 in 40% *v*/*v* alcohol. The results therefore suggest that the differences in the gel layer growth do not significantly affect drug release in this instance. With regard to the mechanisms of drug release, it was observed that there was a drive towards swelling as alcohol content increased, but anomalous transport dominated overall (Table 2). 

Using drug release from pH 6.8 for the CR compacts as a standard showed all the release profiles (including that in pH 6.8 with 40% *v*/*v* absolute ethanol) to be similar (Table 2). The similarity values were lower than those in acidic media. In the case of the DC compacts, the profile at 40% *v*/*v* absolute ethanol content was dissimilar (45.3) when compared to the drug release profile in the absence of alcohol. MRI studies indicated that, although the dry core was more depleted for CR compacts, the CR polymer produced significant gel layer formation, resulting in an increased diffusion pathway for PPN compared to DC compacts. This is also influenced by the less intact gel structure in the presence of alcohol. As the pKa of propranolol is 9.67, the drug should be mainly ionised at both pH levels. Ionic strength is higher in the pH 6.8 media and may cause a decrease in the amount of dissolution medium uptake as a result of a “salting out” effect by inorganic ions in the dissolution media [64]. This causes a loss in the water of hydration for the polymer’s molecular chain as a result of competition for water of hydration [7,64,65]. At this pH, the addition of 40% *v*/*v* absolute ethanol causes further competition for water, hence the significant decreases in drug release (Table 2). Although there is an increased tendency towards swelling controlled release for DC, the differences between the particle properties such as flow, charge and adhesion did not significantly impact release behaviour. They may, of course, have implications for the handling of powders and processes prior to compaction and release.

## 4. Conclusions

The CR and DC grades of hypromellose were evaluated for their particle, flow, tabletting and dissolution properties. The results showed that the difference in morphology for the polymer impacted the charge, adhesion and flow properties, which can impact the manufacturing process. The differences in particle morphology, however, did not significantly impact the mechanical strength and dissolution behaviour of formulations of the model soluble drug. X-ray microtomography and MRI imaging was successfully used as a complementary technique to determine the gel layer/hydration layer measurements as well as ingress of hydroalcoholic media and its impact on the dry core. We were able to determine that, although the inclusion of alcohol did impact the hydration behaviour of the different hypromellose polymers, this also did not impact significantly on the drug release process. 

## Figures and Tables

**Table 1:** Materials used in the formulation of CR and DC compacts used in the study.

Ingredient	Percent (%)
Propranolol HCl	20
Microcrystalline cellulose (MCC)	39
Hypromellose 2208 *	30
Pregelatinised starch (Starch 1500)	10
Colloidal silica	0.5
Magnesium sterate	0.5
Total:	100

* Indicates either CR or DC grade used to allow for comparison.

**Table 2:** Dissolution parameters and mechanism of drug release from CR and DC hypromellose compacts in various hydroalcoholic media.

Formulation Media	T_50_ (min)	DE (%)	MDT (min)	MDR (%min^−1^)	Similarity Factor (f2)	Diffusional Exponent (*n*)
CR-pH 1.2	63.46 (1.52) ^c^	58.56	249.59	0.17	-	0.57
CR-pH 1.2 ^a^	62.15 (0.37)	57.36	252.84	0.16	90.94	0.57
CR-pH 1.2 ^b^	65.28 (1.71)	59.75	235.23	0.17	80.12	0.64
DC-pH 1.2	69.34 (7.26)	64.03	241.75	0.19	-	0.53
DC-pH 1.2 ^a^	65.07 (1.42)	60.23	256.83	0.17	70.05	0.62
DC-pH 1.2 ^b^	66.65 (7.80)	61.5	244.19	0.18	79.28	0.66
CR-pH 6.8	56.91 (2.76)	54.14	245.96	0.16	-	0.5
CR-pH 6.8 ^a^	53.76 (0.49)	50.83	248.82	0.15	73.66	0.53
CR-pH 6.8 ^b^	68.03 (0.59)	62.96	229.38	0.18	54.43	0.63
DC-pH 6.8	50.79 (0.38)	47.66	272.55	0.13	-	0.81
DC-pH 6.8 ^a^	54.18 (2.59)	50.53	265.04	0.14	77.7	0.76
DC-pH 6.8 ^b^	67.18 (2.46)	61.21	239	0.17	45.3	0.87

Note: ^a^ is medium containing 5% *v*/*v* absolute ethanol, ^b^ is medium containing 40% *v*/*v* absolute ethanol, ^c^ Standard deviation.

## Abbreviations

HPMC, hydroxypropyl methylcellulose; MCC, microcrystalline cellulose; PPN, propranolol HCl; MDT, mean dissolution time; MDR, mean dissolution rate; DE, dissolution efficiency; USP, United States Pharmacopeia; API, active pharmaceutical ingredient; MgSt, magnesium stearate; XµT, x-ray microtomography; MRI, magnetic resonance imaging; SEM, scanning electron microscope; CMR, charge to mass ratio; P-CMR, positive charge to mass ratio; N-CMR, negative charge to mass ratio.
